# Gene Expression Profiling of *Pseudomonas aeruginosa* Upon Exposure to Colistin and Tobramycin

**DOI:** 10.3389/fmicb.2021.626715

**Published:** 2021-04-30

**Authors:** Anastasia Cianciulli Sesso, Branislav Lilić, Fabian Amman, Michael T. Wolfinger, Elisabeth Sonnleitner, Udo Bläsi

**Affiliations:** ^1^Max Perutz Labs, Vienna Biocenter (VBC), Department of Microbiology, Immunobiology and Genetics, University of Vienna, Vienna, Austria; ^2^Institute for Theoretical Chemistry, University of Vienna, Vienna, Austria; ^3^Research Group Bioinformatics and Computational Biology, Faculty of Computer Science, University of Vienna, Vienna, Austria

**Keywords:** *Pseudomonas aeruginosa*, colistin, tobramycin, RNA-Seq, ribosome profiling, Ribo-seq

## Abstract

*Pseudomonas aeruginosa* (*Pae*) is notorious for its high-level resistance toward clinically used antibiotics. In fact, *Pae* has rendered most antimicrobials ineffective, leaving polymyxins and aminoglycosides as last resort antibiotics. Although several resistance mechanisms of *Pae* are known toward these drugs, a profounder knowledge of hitherto unidentified factors and pathways appears crucial to develop novel strategies to increase their efficacy. Here, we have performed for the first time transcriptome analyses and ribosome profiling in parallel with strain PA14 grown in synthetic cystic fibrosis medium upon exposure to polymyxin E (colistin) and tobramycin. This approach did not only confirm known mechanisms involved in colistin and tobramycin susceptibility but revealed also as yet unknown functions/pathways. Colistin treatment resulted primarily in an anti-oxidative stress response and in the de-regulation of the MexT and AlgU regulons, whereas exposure to tobramycin led predominantly to a rewiring of the expression of multiple amino acid catabolic genes, lower tricarboxylic acid (TCA) cycle genes, type II and VI secretion system genes and genes involved in bacterial motility and attachment, which could potentially lead to a decrease in drug uptake. Moreover, we report that the adverse effects of tobramycin on translation are countered with enhanced expression of genes involved in stalled ribosome rescue, tRNA methylation and type II toxin-antitoxin (TA) systems.

## Introduction

*Pseudomonas aeruginosa* (*Pae*) is an opportunistic pathogen known to cause nosocomial infections that are particularly detrimental to immunocompromised individuals and to patients suffering from cystic fibrosis (CF) (Williams et al., 2010). On the one hand, the pathogenic potential of *Pae* is based on its metabolic versatility, permitting fast adaptation to changing environmental conditions. On the other hand *Pae* can form biofilms and produce multiple virulence factors (Kerr and Snelling, 2009; Gellatly and Hancock, 2013). *Pae* is characterized by high intrinsic resistance to a wide variety of antibiotics. It can further develop resistance by acquisition of genetic determinants through horizontal gene transfer, as well as by mutational processes affecting “resistance genes” that are collectively termed the resistome (Wright, 2007; Fajardo et al., 2008; Breidenstein et al., 2011; Jaillard et al., 2017). In this way, *Pae* has rendered most antibiotics ineffective, leaving polymyxins and aminoglycosides as last resort antibiotics.

Polymyxins are polycationic cyclic antimicrobial peptides. Owing to their positively charged 2,4-diaminobuteric acid (Dab) moieties they can electrostatically interact with the negatively charged lipopolysaccharide (LPS) of the outer membrane (OM) of Gram-negative bacteria, causing the displacement of LPS-stabilizing divalent cations, Ca^2+^ and Mg^2+^. This interaction is followed by insertion of the hydrophobic segments of the drug into the OM and its penetration *via* a self-promoted uptake mechanism. Cell death subsequently occurs by disintegration of the inner membrane (IM) and leakage of cellular components (El-Sayed Ahmed et al., 2020). Moreover, it has been reported that polymyxins can exert their toxic effects by causing phospholipid exchange between the OM and IM (Velkov et al., 2013), inhibition of respiratory enzymes of the NADH oxidase family (Deris et al., 2014), binding to bacterial DNA and disrupting its synthesis (Kong et al., 2011) and/or formation of reactive oxygen species (ROS) (Sampson et al., 2012; Yu et al., 2017). Nevertheless, the mode of bactericidal action of polymyxins in *Pae* remains controversial. For instance, a recent study (O’Driscoll et al., 2018) indicated that polymyxin E (colistin) does not exert its antibacterial effect by puncturing the IM or by inhibiting DNA replication and transcription. In addition, the exact contribution of polymyxin induced ROS to lethality of *Pae* is largely inconclusive (Brochmann et al., 2014; Lima et al., 2019).

In contrast, the regulatory circuits underlying polymyxin resistance are well understood in *Pae*. An increased resistance is conveyed by reduction of the net negative charge of LPS, resulting in diminished polymyxin binding (Jeannot et al., 2017; Poirel et al., 2017). The cellular machinery for covalent modification of negatively charged lipid A of LPS with positively charged 4-amino-L-arabinose (Lara4N) is encoded by the *arn* (*pmr*) operon. This operon is activated by at least five two-component systems (TCS) including PhoP/PhoQ (Barrow and Kwon, 2009), PmrA/PmrB (McPhee et al., 2003), ParR/ParS (Fernández et al., 2010), ColR/ColS and CprR/CprS (Fernández et al., 2012; Gutu et al., 2013). In addition, the *cprA* gene product was found to be required for polymyxin resistance conferred by the PhoP/PhoQ, PmrA/PmrB, and CprR/CprS TCSs (Gutu et al., 2013). Furthermore, a number of other functions contributing to intrinsic polymyxin resistance have been identified, which mainly affect LPS biosynthesis-related functions (regulatory functions, metabolism, synthesis and transport) (Fernández et al., 2013; Zhang et al., 2017; Sherry and Howden, 2018). Moreover, overproduction of spermidine and of the OM protein OprH have been shown to contribute as well to polymyxin susceptibility, as they can interact with divalent cation-binding sites of LPS, making them inaccessible for polymyxin binding (Young et al., 1992; Johnson et al., 2011). On the other hand, a reduced expression of *oprD* increased cell survival in the presence of polymyxins through an unknown mechanism (Mlynarcik and Kolar, 2019). Additionally, the MexXY-OprM and MexAB-OprM efflux pump systems can provide low to moderate polymyxin resistance and tolerance respectively (Pamp et al., 2008; Muller et al., 2011; Poole et al., 2015).

Aminoglycosides are positively charged antibiotics that initially interact with LPS of Gram-negative Bacteria. Aminoglycosides require an energized membrane for translocation into the cytoplasm. Once inside the cells, they bind to 16S rRNA at the A-site of the 30S ribosomal subunit, disrupting translation and causing the synthesis of aberrant polypeptides. These polypeptides can be inserted into the cell membrane, causing membrane damage, which leads to further intracellular accumulation of aminoglycosides. The established autocatalytic loop of membrane damage and their increased uptake results in stalling of ribosomes, and in complete inhibition of protein synthesis (Krause et al., 2016).

Covalent modifications of the negatively charged moieties of LPS, 16S rRNA methylation by RNA methyltransferases, ribosomal mutations and aminoglycoside modifying enzymes (AMEs) are exploited by *Pae* to counteract aminoglycosides (Poole, 2005; Garneau-Tsodikova and Labby, 2016; Krause et al., 2016; Valderrama-Carmona et al., 2019). The main efflux system responsible for the extrusion of aminoglycosides is MexXY-OprM (Poole, 2005). Its synthesis is controlled by PA5471, an anti-repressor of the *mexXY* operon repressor MexZ (Morita et al., 2006; Hay et al., 2013). Additional efflux systems include MexAB-OprM and an ortholog of the EmrE multidrug transporter of *Escherichia coli* (Poole, 2005; Nasie et al., 2012). Moreover, protein chaperones such as GroEL/ES, GrpE and HtpX, as well as the AmgR/AmgS TCS have been implicated in protecting the cells from polypeptides arising from drug induced mistranslation (Hinz et al., 2011; Wu et al., 2015).

A number of studies have confirmed the safety of colistin for treatment of acute pulmonary infections, while tobramycin was proven effective in suppressing chronic *Pae* airway infections in CF patients (Ramsey et al., 1999; Garnacho-Montero et al., 2003). However, in recent years a gradual decrease in baseline susceptibility of *Pae* to these last resort antibiotics was observed (Obritsch et al., 2004; Wi et al., 2017; Jain, 2018). As a refined understanding of the molecular regulatory circuits that contribute to resistance, tolerance and persister cell formation is key to develop new strategies/tools to combat *Pae*, we have employed RNA-seq and Ribo-seq in parallel to monitor gene expression responses of the clinical *Pae* isolate PA14 grown in synthetic cystic fibrosis sputum medium (SCFM) to inhibitory concentrations of colistin and tobramycin.

In addition to *arn* operon activation, which is known to result in reduced drug uptake, *Pae* responds to colistin by launching an anti-oxidative response, and by de-regulating genes belonging to the MexT and AlgU regulons. Concerning tobramycin, *Pae* seemingly goes through metabolic changes and envelope remodeling to prevent drug uptake, whereas its ramifications on translational processes are met with the stalled ribosome rescue response and the activation of type II toxin-antitoxin (TA) systems.

## Materials and Methods

### Bacterial Strains and Growth Conditions

The clinical isolate *Pae* PA14 (Rahme et al., 1995) was used in all gene expression profiling experiments. Synthetic cystic fibrosis sputum medium (SCFM) was prepared as previously described (Palmer et al., 2007) with the modification specified in Tata et al. (2016). PA14 cells were grown aerobically in 500 ml SCFM at 37°C. At an OD_600_ of 1.7, the cultures were treated with inhibitory concentrations of colistin (8 μg/ml; Sigma) and tobramycin (64 μg/ml; Sigma), respectively, or water was added as a control. The cultures reached OD_600_ of 2 approximately 2 h after exposure to the antibiotics, as can be inferred from Supplementary Figure 1. 10 ml samples were withdrawn for RNA-seq analyses, while the remaining culture volume was used for the Ribo-seq experiments. The strain PA14*ΔalgU* was constructed as described in the Supplementary Text.

### RNA-Seq

Total RNA was isolated from two biological replicates using the Trizol method (Ambion) according to the manufacturer’s instructions. The samples were treated with DNase I (TURBO^TM^ DNase, Thermo Scientific), followed by phenol-chloroform-isoamyl alcohol (25:24:1) extraction and ethanol precipitation. Ribosomal RNA was depleted with The Ribo-Zero^TM^ rRNA Removal Kit. The libraries were constructed using the NEBNext^®^ Ultra^TM^ Directional RNA Library Prep Kit for Illumina^®^. Hundred bp single end sequence reads were generated using the Illumina HiSeq200 platform at the in house Next Generation Sequencing Facility (VBCF, Vienna, Austria^1^). Quality control assessment of the raw reads using FastqQC^2^ obviated further pre-processing. Sequencing adapter removal was performed with cutadap (Martin, 2011). Mapping of the samples against the PA14 reference genome (NCBI accession number NC_008463.1) was performed with Segemehl (Hoffmann et al., 2009) with default parameters. Reads mapping to rRNA or tRNA genes were discarded from all data and ignored for all follow-up analyses. The mapped sequencing data were prepared for visualization using the ViennaNGS tool box and visualized with the UCSC Genome Browser (Wolfinger et al., 2015). Reads per gene were counted using BEDTools (Quinlan and Hall, 2010) and the Refseq annotation of *Pae* (NC_002516.2). Differential gene expression analysis was performed with DESeq (Anders and Huber, 2010). All genes with a fold-change (FC) greater than ±2 and a multiple testing adjusted *p*-value below 0.05 were considered to be significantly modulated. The raw sequencing data were deposited in the European Nucleotide Archive (ENA) under accession number PRJEB41029.

### Ribo-Seq

Ribosome profiling of elongating ribosomes (Ribo-seq; Ingolia et al., 2009) was performed with the same cultures as used for the RNA-seq analyses. Upon culture growth, the cells were treated for 10 min with chloramphenicol (300 μg/ml) to stop translation, and then harvested by centrifugation at 8,000 *g* for 15 min at 0°C. The cells were washed in 50 ml ice cold lysis buffer (10 mM MgOAc, 60 mM NH_4_Cl, 10 mM TRIS-HCl, pH 7.6) and again pelleted by centrifugation at 5000 *g* for 15 min at 4°C. The pellets were re-suspended in 1 ml ice cold lysis buffer containing 0.2% Triton X-100, 100 μg/ml chloramphenicol and 100 U/ml DNAse I, frozen in liquid nitrogen and cryogenically pulverized by repeated cycles of grinding in a pre-chilled mortar and freezing in a dry ice/ethanol bath. These lysates were centrifuged at 15,000 *g* for 30 min at 4°C to remove cellular debris. Hundred μl aliquots of the cleared lysates were treated with 4 μl of Micrococcal Nuclease (MNase, NEB) and 6 μl of the RiboLock RNase inhibitor (Thermo Scientific) for 1 h at 25°C with continuous shaking at 450 rpm. The lysates were then layered onto 10–40% linear sucrose density gradients in lysis buffer and centrifuged at 256,000 *g* for 3 h at 4°C. Five hundred μl gradient fractions were collected by continuously monitoring the absorbance at 260 nm. The RNA was extracted from fractions containing 70S ribosomes with phenol-chloroform-isoamyl alcohol (25:24:1), and precipitated with ethanol. The samples were then treated with DNase I (TURBO^TM^ DNase, Thermo Scientific) and separated on a 15% polyacrylamide gel containing 8M urea. Ribosome protected mRNA fragments (ribosomal footprints) ranging in size of 20–40 nucleotides were removed and eluted from the polyacrylamide gel by overnight incubation in elution buffer (0.3 M NaOAc, 1 mM EDTA) at 4°C, which was followed by an additional round of phenol-chloroform-isoamyl alcohol (25:24:1) extraction and ethanol precipitation. The quality of RNA samples was subsequently analyzed with a 2100 Bioanalyzer and an Aligned RNA 6000 Pico Kit (Aligned Technologies). The RNA was further processed into cDNA libraries with NEBNext^TM^ Small RNA Library Prep Set for Illumina^®^ and their quality was assessed with the 2100 Bioanalyzer and a High Sensitivity DNA Kit (Agilent Technologies). Pipin Prep^TM^ was used to purify the 140–160 bp cDNA products which corresponded to adapter-ligated 20–40 nucleotide long ribosomal footprints. RNA sequencing and data processing was performed as described above. The raw sequencing data were deposited in the ENA under accession number PRJEB41027.

## Results and Discussion

### Quality Assessment and Data Analysis

**FIGURE 1:**
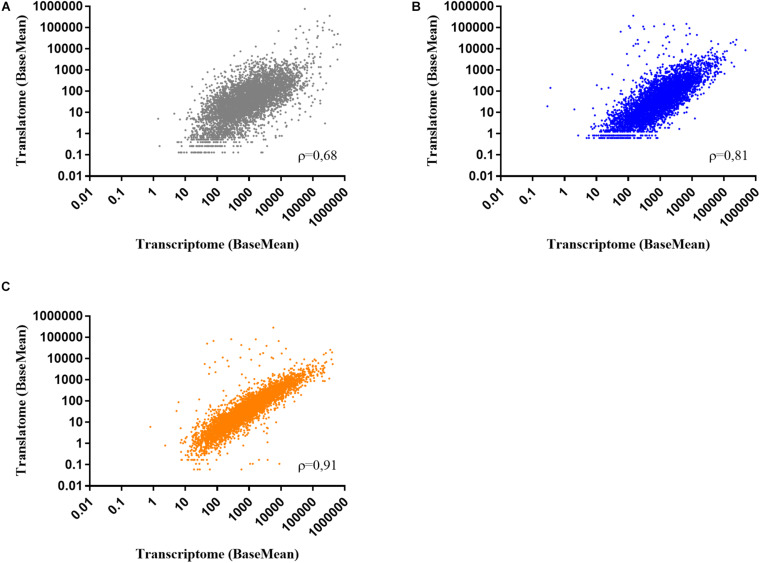
Scatter plots showing the correlation between normalized RNA-seq and Ribo-seq sequencing reads (BaseMean) obtained from two biological replicates of **(A)** control, **(B)** colistin, and **(C)** tobramycin treated samples. ρ – Spearman correlation value.

To determine the effect of colistin and tobramycin on gene expression, parallel RNA-seq and Ribo-seq experiments were performed with planktonically grown PA14. The cultures reached OD_600_ of 2 approximately 2 h after exposure to the antibiotics, as can be inferred from Supplementary Figure 1. As a control, total RNA and ribosome protected mRNA fragments (ribosomal footprints) were isolated from cultures grown without antibiotics. As the number of ribosomal footprint sequencing reads have been shown to correlate with those obtained from RNA-seq experiments (Ingolia et al., 2009), we first determined the representative gene expression correlations between RNA-seq and Ribo-seq. The number of RNA-seq and Ribo-seq sequencing reads were normalized (BaseMean), and the Spearman correlation value (ρ-value) between them was assessed for each condition (controls, colistin and tobramycin treatment). The correlation coefficient between the average Ribo-seq and RNA-seq BaseMean expression values was 0.68 for the control, 0.81 for colistin, and 0.91 for tobramycin treated samples, respectively (Figure 1). Similar ρ-values have been also reported by other studies (Blevins et al., 2018).

**FIGURE 2:**
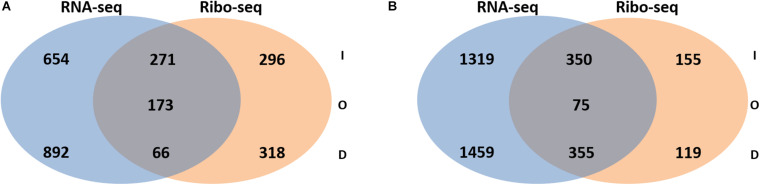
Venn diagram showing the number of transcripts with increased (I), decreased (D) or opposite (O) abundance in RNA-seq and Ribo-seq data obtained after **(A)** colistin treatment and **(B)** tobramycin treatment. For significance, only transcripts with a fold-change ≥ 2 or ≤ –2 and a multiple testing adjusted *p*-value ≤ 0.05 were considered. The corresponding transcripts and ribosomal footprints with increased, decreased and opposite abundance are listed in Supplementary Table 1.

**FIGURE 3:**
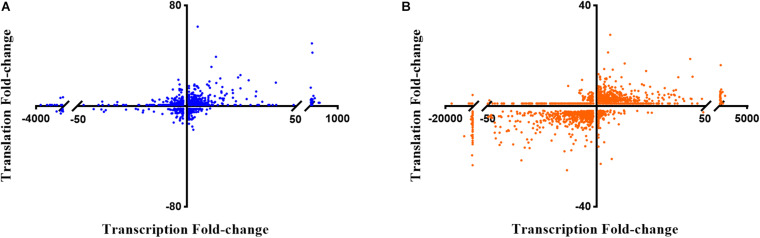
Scatter plots showing the correlation between gene expression fold-changes in RNA-seq and Ribo-seq data obtained after **(A)** colistin treatment and **(B)** tobramycin treatment. The *X*-axis corresponds to the RNA-seq data, or transcriptome, and the *Y*-axis to the Ribo-seq data, or translatome.

**FIGURE 4:**
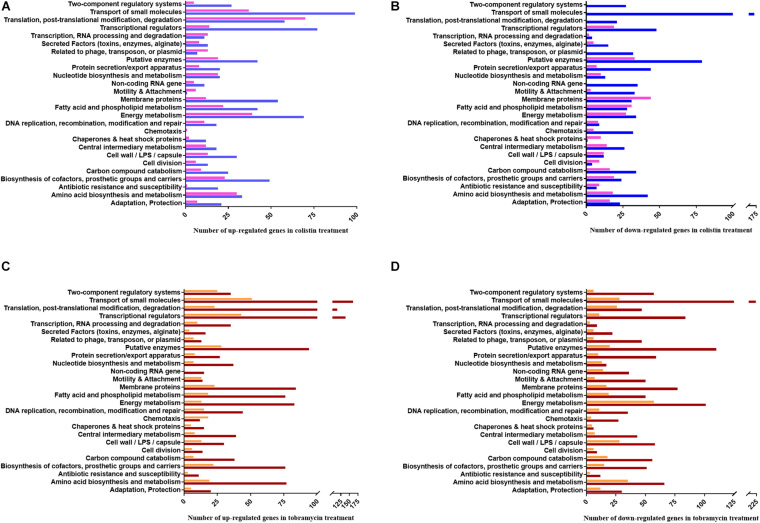
PseudoCAP functional class distribution of annotated genes with altered expression in response to colistin or tobramycin. **(A)** Up-regulated and **(B)** down-regulated genes in colistin treated samples. Blue and pink bars indicate the number of de-regulated genes based on the RNA-seq and Ribo-seq data, respectively. **(C)** Up-regulated and **(D)** down-regulated genes in tobramycin treated samples. Brown and orange bars indicate the number of de-regulated genes based on the RNA-seq and Ribo-seq data, respectively.

Next, the FC in transcript abundance between antibiotic treated and untreated samples was calculated. The following criteria were applied for differential gene expression analysis and interpretation: (i) only annotated genes deposited in the *Pseudomonas* genome database (Winsor et al., 2016) were considered for comparison; (ii) genes with a low expression level (less than 100 RNA-seq or 50 Ribo-seq reads) were disregarded; (iii) for all data sets a p-value (adjusted for multiple testing) of 0.05 was set as a threshold for significance and (iv) the change in FC had to exceed ± 2 for a given gene to be regarded as differentially expressed. When compared with the sequencing data acquired from the non-treated samples, 2056 and 3558 genes were found to be differentially abundant in RNA-seq after exposure to colistin and tobramycin, respectively, whereas that number in Ribo-seq amounted to 1124 and 1045 (Figure 2 and Supplementary Table 1). The scatter plots depicting the correlation between RNA-seq and Ribo-seq gene FC values are shown in Figure 3. Discrepancies in the number of de-regulated genes in RNA-seq when compared to Ribo-seq data have been reported before (Blevins et al., 2018), highlighting the importance of parallel application of these methods for assessment of gene expression. Interestingly, the vast majority of genes were significantly differentially expressed solely at the transcriptional or at the translational level by colistin and tobramycin. 1546 genes were de-regulated by colistin exclusively at the transcriptional level, whereas 614 genes were only affected at the translational level. In case of tobramycin 2778 genes showed FC values that exceeded ± 2 only in the RNA-seq data, while 274 genes were differentially expressed only in the Ribo-seq data. Moreover, 173 and 75 transcripts displayed opposite FC values in the two data sets after treatment with colistin and tobramycin, respectively (Figure 2 and Supplementary Table 1). These results showed that the differentially abundant transcripts observed with RNA-seq did not highly correlate with the outcome of the Ribo-seq analyses and *vice versa*. An explanation for this observation could be that the expression of these genes is post-transcriptionally regulated. In any case, the patterns of PseudoCAP functional class distribution of annotated transcripts with altered expression in response to colistin or tobramycin were similar for the transcriptome and translatome data (Figure 4).

### Known Gene Expression Responses to Colistin and Tobramycin

To validate our data, we first scrutinized an assortment of genes known to be involved in maintenance of intrinsic and/or adaptive resistance of *Pae* toward colistin and tobramycin. In the case of colistin, we assessed the expression levels of the *oprD*, *pmrA*, and *pmrB* transcripts and of genes involved in the synthesis (i) and modification of LPS (such as the *arn* operon, *pagL*, *lpxO2*, *lpxC*, and *galU*), (ii) of spermidine (*PA14_63110 – PA14_63120*), (iii) of the short-chain dehydrogenase/reductase family protein CprA (PA14_43311) and (iv) of the MexXY (PA14_38395-AmrB) and MexAB-OprM efflux pumps. As anticipated, the above mentioned genes were up-regulated upon colistin treatment, with the exception of *oprD* whose expression was down-regulated (Supplementary Table 2).

In the case of tobramycin, the abundance of genes known to be involved in (i) drug modification (*aph*), (ii) target binding inhibition (*rsmE*), (iii) extrusion (*mexXY* operon anti-repressor PA14_72210), (iv) maintenance of the cell membrane (*groEL/ES, grpE*, and *htpX*) as well as the genes encoding the AmgR/AmgS (OmpR/EnvZ) TCS were scrutinized. The transcription and/or translation of all the above mentioned genes was enhanced upon tobramycin treatment (Supplementary Table 2).

At a glance, energy metabolism-, translation-, and transcription- functional classes of genes were up-regulated after colistin exposure. On the other hand, colistin appears to negatively affect the abundance of mRNAs encoding functions involved in transport of small molecules, motility and attachment. Moreover, it translationally impaired expression of membrane protein genes (Figure 4).

The functional classes representing the majority of predominantly positively affected genes by tobramycin are related to transcriptional regulators, RNA processing and translation, whereas the most down-regulated gene functions are involved in energy metabolism, carbon compound catabolism and cell wall/LPS/capsule synthesis. Interestingly, motility and attachment genes were prominently down-regulated by tobramycin at the transcriptional level, whereas amino acid biosynthesis and metabolism genes were apparently more negatively affected at the translational level (Figure 4).

To find additional players and pathways involved in colistin and/or tobramycin resistance in *Pae*, we next took a closer look at all genes which displayed a ±10 FC in transcript abundance in antibiotic treated samples in the RNA-seq and/or Ribo-seq data sets (Supplementary Table 1).

### Colistin Induces Oxidative Stress Response Genes

The accepted mode of action of polymyxins, i.e., causing a lesion in the IM, has been challenged by the finding that even supra-bactericidal colistin concentrations induced minor loss of intracellular components (O’Driscoll et al., 2018). However, polymyxins are also known to elicit oxidative damage in Bacteria through the production of ROS, such as superoxide O_2_^–^, hydrogenperoxide H_2_O_2_ and hydroxy radicals ⋅OH (El-Sayed Ahmed et al., 2020). Both O_2_^–^ and H_2_O_2_ can injure proteins that possess iron–sulfur ([Fe–S]) clusters as cofactors. The maintenance of [Fe–S] proteins is of importance as they are required for many biological processes, including protein biosynthesis, respiration, central metabolism, photosynthesis, nitrogen fixation, DNA repair, RNA modification and gene regulation (Roche et al., 2013; Kimura and Suzuki, 2015). Polymyxin induced oxidative damage has been reported for Gram-negative and Gram-positive species, including *Acinetobacter boumannii* (Sampson et al., 2012), *Pae* (Brochmann et al., 2014; Lima et al., 2019), *Bacillus subtilis*, and the natural producer *Paenibacillus polymyxa* (Yu et al., 2019). Studies performed on the Gram-positive *P. polymyxa* provided a detailed explanation of how polymyxins might lead to ROS production. It has been hypothesized that colistin stimulates the tricarboxylic acid (TCA) cycle through an increase in the production of isocitrate (*icdA*), α-ketoglutaric (*sucB*), and malate (*mdh*) dehydrogenases, which in turn leads to increased NADH production and enhanced respiration rates (Yu et al., 2019). Accordingly, the concentration of O_2_^–^ surges intracellularly, where it can be converted to H_2_O_2_ by the superoxide dismutase (SOD). The *sodA* (Mn-SOD) and *sodB* (Fe-SOD) genes were up-regulated in *P. polymyxa* in the presence of colistin (Yu et al., 2017), while inactivation of *sodB* in the Gram-negative bacterium *A. boumannii* augmented its susceptibility to the same drug (Heindorf et al., 2014). Moreover, the involvement of *sodC* (CuZn-SOD) and catalase encoding *katA* genes in polymyxin resistance was observed in *A. boumannii* and *Staphylococcus aureus*, respectively (Antonic et al., 2013; Pournaras et al., 2014).

In our study, exposure of *Pae* to inhibitory concentration of colistin resulted in an up-regulation of genes involved in the oxidative stress response (Table 1 and Supplementary Table 1). These genes include *aphF* (Ochsner et al., 2000b), *iscR* (*PA14_14710*) (Romsang et al., 2014), the *PA14_21570*-*PA14_21580-PA14_21590-PA14_21600* operon (Farrant et al., 2020), and *PA14_22320* (Salunkhe et al., 2005). Next, we assessed whether additional genes required for alleviation of ROS were differentially expressed upon colistin treatment, but were initially not accounted for due to the set ±10 FC threshold. The catalase encoding *katA* and *katB* genes (Brown et al., 1995; Ma et al., 1999), as well as the gene encoding their regulator OxyR (Wei et al., 2012) were up-regulated at both the transcriptional and translational level (Table 1 and Supplementary Table 1). However, the alkyl hydroperoxide reductase gene *ahpB* (Ochsner et al., 2000b) and the superoxide dismutase gene *sodB* (Hassett et al., 1993, 1995) were only found to be up-regulated in the RNA-seq data (Table 1 and Supplementary Table 1). Moreover, the *soxR* gene and the majority of genes regulated by the redox-responsive SoxR regulator (*mexG, mexH, mexI, PA14_16310*, and *PA14_35160*) (Palma et al., 2005) displayed an increased transcript and ribosomal footprint abundance (Table 1 and Supplementary Table 1).

**TABLE 1:** Gene expression response of PA14 grown in the presence of colistin versus untreated control.

**Gene name**	**Gene ID**	**Gene product**	**RNA-seq**	**Ribo-seq**
			**FC^1^**	**FC**
**Oxidative stress response genes**				
**ahpF**	PA14_01720	Alkyl hydroperoxide reductase	**52.54**^2^	4.99
**katA**	PA14_09150	Catalase	8.45	4.75
**mexI**	PA14_09520	RND efflux transporter	2.31	2.5
**mexH**	PA14_09530	RND efflux membrane fusion protein	3.36	ND^3^
**mexG**	PA14_09540	Hypothetical protein	4.7	4
**PA14_14710**	PA14_14710	Rrf2 family protein	9.31	**17.2**
**PA14_16310**	PA14_16310	MFS permease	**22.54**	3.98
**PA14_21570**	PA14_21570	Hypothetical protein	**12.84**	8.05
**PA14_21580**	PA14_21580	Hypothetical protein	**15.15**	6.37
**PA14_21590**	PA14_21590	Hypothetical protein	**10.72**	9
**PA14_21600**	PA14_21600	Hypothetical protein	9.95	6.61
**PA14_22320**	PA14_22320	Hypothetical protein	**10.1**	3.97
**PA14_35160**	PA14_35160	Hypothetical protein	4.89	2.74
**soxR**	PA14_35170	Redox-sensing activator of *soxS*	3.86	3.02
**PA14_53300**	PA14_53300	Alkyl hydroperoxide reductase AhpB	**106.45**	ND
**sodB**	PA14_56780	Cation-transporting P-type ATPase	9.57	ND
**katB**	PA14_61040	Superoxide dismutase	9.26	2.91
**oxyR**	PA14_70560	LysR family transcriptional regulator	2.43	2.72
**Iron homeostasis genes**				
**exbB1**	PA14_02500	Transport protein ExbB	–2.93	X^4^
**exbD1**	PA14_02510	Transport protein ExbD	**–10.34**	X
**pchA**	PA14_09210	Salicylate biosynthesis isochorismate synthase	–3.35	**17.28**
**pchB**	PA14_09220	Isochorismate-pyruvate lyase	–3.09	**13.26**
**pchC**	PA14_09230	Pyochelin biosynthetic protein PchC	–2.35	6.34
**pchD**	PA14_09240	Pyochelin biosynthesis protein PchD	–6.01	ND
**pchR**	PA14_09260	Transcriptional regulator PchR	ND	4.48
**pchE**	PA14_09270	Dihydroaeruginoic acid synthetase	ND	6.47
**pchF**	PA14_09280	Pyochelin synthetase	–2.56	**11.04**
**pchG**	PA14_09290	Pyochelin biosynthetic protein PchG	–2.25	**13.01**
**pchH**	PA14_09300	ABC transporter ATP-binding protein	–3.52	9.05
**pchI**	PA14_09320	ABC transporter ATP-binding protein	–2.91	**13.46**
**fptA**	PA14_09340	Fe(III)-pyochelin outer membrane receptor	–4.70	7.57
**PA14_20000**	PA14_20000	Transmembrane sensor	**–11.29**	X
**hasR**	PA14_20010	Heme uptake outer membrane receptor HasR	**–32.17**	X
**hasAp**	PA14_20020	Heme acquisition protein HasAp	**–1282.44**	X
**hasD**	PA14_20030	Transport protein HasD	**–38.16**	X
**hasE**	PA14_20040	Metalloprotease secretion protein	**–56.02**	X
**hasF**	PA14_20050	Outer membrane protein	**–27.96**	X
**pvdS**	PA14_33260	Extracytoplasmic-function sigma-70 factor	**–12.48**	X
**pvdG**	PA14_33270	Protein PvdG	–5.48	X
**pvdL**	PA14_33280	Peptide synthase	**–15.82**	X
**PA14_33420**	PA14_33420	Hydrolase	–1.98	X
**PA14_33610**	PA14_33610	Peptide synthase	**–14.69**	X
**pvdJ**	PA14_33630	Protein PvdJ	**–31.99**	X
**pvdD**	PA14_33650	Pyoverdine synthetase D	**–16.41**	X
**fpvA**	PA14_33680	Ferripyoverdine receptor	–5.16	X
**pvdE**	PA14_33690	Pyoverdine biosynthesis protein PvdE	–4.05	X
**pvdF**	PA14_33700	Pyoverdine synthetase F	–2.71	X
**pvdO**	PA14_33710	Protein PvdO	**–10.04**	X
**pvdN**	PA14_33720	Protein PvdN	–6.41	X
**PA14_33730**	PA14_33730	Dipeptidase	–3.91	X
**opmQ**	PA14_33750	Outer membrane protein	–4.9	X
**PA14_33760**	PA14_33760	ABC transporter ATP-binding protein/permease	–3.12	X
**PA14_33780**	PA14_33780	Transmembrane sensor	–1.89	X
**pvdA**	PA14_33810	L-ornithine N5-oxygenase	**–10.84**	X
**pvdQ**	PA14_33820	Penicillin acylase-related protein	–8.57	X
**feoC**	PA14_56670	Hypothetical protein	–9.56	X
**feoB**	PA14_56680	Ferrous iron transport protein B	**–38.95**	X
**feoA**	PA14_56690	Ferrous iron transport protein A	**–68.23**	X
**MexT regulon genes**				
**PA14_22420**	PA14_22420	Hypothetical protein	**10.58**	**14.6**
**PA14_22740**	PA14_22740	Hypothetical protein	**20.99**	**12.81**
**PA14_28410**	PA14_28410	Hypothetical protein	**21.19**	**10.47**
**mexF**	PA14_32390	RND multidrug efflux transporter MexF	2.28	4.37
**mexE**	PA14_32400	RND multidrug efflux membrane fusion protein MexE	9.8	4.98
**PA14_32480**	PA14_32480	Hypothetical protein	3.72	4.726
**PA14_32490**	PA14_32490	Hypothetical protein	5.96	5.49
**PA14_39060**	PA14_39060	Hypothetical protein	**28.44**	**20.56**
**PA14_39420**	PA14_39420	Hypothetical protein	**18.31**	9.99
**PA14_41990**	PA14_41990	Hypothetical protein	2.26	**10.38**
**PA14_56620**	PA14_56620	Hypothetical protein	**31.66**	7.99
**PA14_56640**	PA14_56640	MFS transporter	**27.52**	5.7
**PA14_64530**	PA14_64530	Hypothetical protein	**195.2**	6.35
**Anaerobic respiratory chain genes**				
**nirN**	PA14_06650	c-type cytochrome	**70.18**	ND
**nirE**	PA14_06660	Uroporphyrin-III c-methyltransferase	**69.22**	2.2
**nirJ**	PA14_06670	Heme d1 biosynthesis protein NirJ	**41.09**	ND
**nirH**	PA14_06680	Hypothetical protein	**39.43**	ND
**nirG**	PA14_06690	Transcriptional regulator	**49.08**	ND
**nirL**	PA14_06700	Heme d1 biosynthesis protein NirL	**37.8**	ND
**PA14_06710**	PA14_06710	Transcriptional regulator	**29.29**	ND
**nirF**	PA14_06720	Heme d1 biosynthesis protein NirF	**21.94**	ND
**nirC**	PA14_06730	c-type cytochrome	**62.8**	ND
**nirM**	PA14_06740	Cytochrome c-551	**67.09**	ND
**nirS**	PA14_06750	Nitrite reductase	**24.69**	ND
**nirQ**	PA14_06770	Regulatory protein NirQ	**11.98**	ND
**nirO**	PA14_06790	Cytochrome *c* oxidase subunit	**39.7**	ND
**PA14_06800**	PA14_06800	Hypothetical protein	**119.94**	5.39
**norC**	PA14_06810	Nitric-oxide reductase subunit C	**87.15**	3.2
**norB**	PA14_06830	Nitric-oxide reductase subunit B	**134.06**	3.35
**norD**	PA14_06840	Dinitrification protein NorD	**328.15**	2.77
**narK1**	PA14_13750	Nitrite extrusion protein 1	**–59.67**	X
**narK2**	PA14_13770	Nitrite extrusion protein 2	**–22.48**	X
**narG**	PA14_13780	Respiratory nitrate reductase alpha subunit	–2.33	X
**nosL**	PA14_20150	NosL protein	**56.67**	3.16
**nosY**	PA14_20170	NosY protein	**94.96**	3.06
**nosF**	PA14_20180	NosF protein	**93.83**	3.93
**nosD**	PA14_20190	Copper ABC transporter periplasmic substrate-binding protein	**34.68**	3.5
**nosZ**	PA14_20200	Nitrous-oxide reductase	**61.22**	ND
**nosR**	PA14_20230	Regulatory protein NosR	**73**	ND
**anr**	PA14_44490	Transcriptional regulator Anr	–2.36	–2.32
**Efflux pump genes**				
**mexJ**	PA14_16800	Efflux transmembrane protein	**15.43**	**22.07**
**mexK**	PA14_16820	Efflux transmembrane protein	6.19	**12.07**
**oprJ**	PA14_60820	Outer membrane protein OprJ	6.19	X
**mexD**	PA14_60830	Multidrug efflux RND transporter MexD	4.32	7.02
**mexC**	PA14_60850	Multidrug efflux RND membrane fusion protein	5.32	**14.65**
**Genes known to be up-regulated by polymyxins or in polymyxin**				
**resistant *Pae* strains**				
**PA14_24360**	PA14_24360	Hypothetical protein	**13.58**	**18.41**
**PA14_34170**	PA14_34170	Hypothetical protein	**95.8**	**49.9**
**PA14_41280**	PA14_41280	Beta-lactamase	**111.9**	**42.62**
**PA14_41290**	PA14_41290	Hypothetical protein	**144.84**	X
**PA14_63220**	PA14_63220	Hypothetical protein	**13.35**	**39.33**

*^1^FC, fold-change, *p*-value ≤ 0.05. ^2^Genes with FC ≤ –10 or ≥10 are represented in bold. ^3^ND, not differentially expressed, –2 ≤ FC ≤ 2 and/or *p*-value ≥ 0.05. ^4^X, not efficiently translated in the control and antibiotic treated samples, Ribo-seq BaseMean ≤ 50.*

The toxicity of ROS-generating agents is magnified by ferrous ions (Fe^2+^) through the Fenton reaction (Kohanski et al., 2007; Yeom et al., 2010), wherein H_2_O_2_ is oxidized by Fe^2+^ to generate OH radicals. These can inactivate enzymes and cause DNA and membrane damage, leading to growth arrest and ultimately to cell death (Yeom et al., 2010). Thus, Bacteria generally establish a tight control on expression of iron homeostasis genes. For instance, in *P. polymyxa* the levels of the transcriptional regulator Fur, which represses iron acquisition genes, are increased upon colistin treatment (Yu et al., 2017). Fe^2+^ can be directly taken up from environment or it can be generated through reduction of free intracellular ferric (Fe^3+^) ions bound to siderophores such as pyoveridine (PVD) or pyochelin (PCH), to iron-sulfur ([Fe–S]) cluster proteins or to heme (Ochsner et al., 2000a; Ratledge and Dover, 2000; Wandersman and Delepelaire, 2004; Cartron et al., 2006). Therefore, we also scrutinized the levels of transcripts encoding genes for iron acquisition and storage upon exposure to colistin. As judged from the RNA-seq data, the genes required for PVD biosynthesis and transport (Lamont and Martin, 2003), for heme uptake (*has* locus; Ochsner et al., 2000a), the Feo system of Fe^2+^ uptake and the TonB2-ExbB1-ExbD1 complex (Zhao and Poole, 2000), which serves as an energy coupler for active iron transport across the outer membrane, were down-regulated (Table 1 and Supplementary Table 1). In addition, the majority of these genes was apparently not efficiently translated in neither the control nor in the colistin treated samples (≤50 Ribo-seq reads). Visual inspection of their sequencing profiles in the UCSC Genome Browser (Wolfinger et al., 2015) revealed a very low ribosomal coverage (Supplementary Figure 2), which might be caused by the applied iron rich culturing conditions. For example, siderophore synthesis in *Pseudomonas* sp. is fully inhibited at >4–10 μM iron (Meyer, 1978; Dumas et al., 2013), a concentration far below of what was used in our experimental setup (100 μM FeSO_4_). Counterintuitively, the genes for pyochelin synthesis and uptake are apparently translated in the presence of colistin (Table 1 and Supplementary Table 1). As pyochelin has a weaker affinity for iron when compared with pyoveridine (Dumas et al., 2013), ongoing synthesis could be necessary to meet sufficient metabolic requirements for iron.

In most Gram-negative Bacteria the ferric uptake regulator Fur complexed with Fe^2+^ is responsible for preventing the synthesis of PVD and PCH in iron replete conditions (Ochsner and Vasil, 1996; Vasil and Ochsner, 1999). Analogously to *P. polymixa*, *fur* was slightly up-regulated in both the RNA-seq and Ribo-seq data (Supplementary Table 1). Therefore, an explanation for the apparent translation of the PCH genes remains elusive.

An additional link between colistin resistance and iron homeostasis can be found in the increased synthesis of PA14_04180 (Table 1 and Supplementary Table 1), a putative periplasmic protein with a bacterial oligonucleotide/oligosaccharide-binding (OB-fold) domain, which can bind cationic ligands (Ginalski et al., 2004). Gene *PA14_04180* was found to be regulated by the calcium responsive TCS CarS/CarR and the ferrous iron responsive BqsS/BqsR TCS (Kreamer et al., 2012; Guragain et al., 2016). The BqsS/BqsR system contributes to cationic stress tolerance as it is regulating the expression of several genes with known or predicted functions in polyamine biosynthesis/transport or polymyxin resistance in *Pae* (i.e., *arnB*, *oprH*, and *PA14_63110*) (Kreamer et al., 2015). Moreover, a periplasmic OB-fold protein OmdA, similar to PA14_04180, is controlled by the PmrA/PmrB TCS and was found to confer resistance to polymyxin B (Pilonieta et al., 2009).

In contrast to *P. polymyxa* (Yu et al., 2019), we did not notice an up-regulation of TCA cycle genes or drastic changes in expression of the NADH oxidase family genes (Supplementary Table 1). Alternatively, it has been hypothesized that O_2_^–^ production could be induced in Gram-negative Bacteria during transit of polymyxin molecules through the cell envelope (Kohanski et al., 2007; El-Sayed Ahmed et al., 2020) and *via* inhibition of type II NADH-quinone oxidoreductases (NDH-2) (Deris et al., 2014).

### Denitrification Pathway Genes Are De-Regulated in the Presence of Colistin

The transcripts encoding for enzymes required for the denitrification pathway (Schreiber et al., 2007), which include the nitrite reductase encoding *nir*-operon, the nitric oxide reductase encoding *nor*-operon and the nitrous dioxide reductase encoding *nos*-operon, were highly abundant relative to their representation in cells growing in the absence of colistin. Similar trends in expression were observed in the Ribo-seq data set, albeit not to the same degree (Table 1 and Supplementary Table 1). Surprisingly, the master regulator of the denitrification pathway ANR and the genes of the *nar*-operon, which encode nitrate reductase, a complex that catalyzes the first step of denitrification, were down-regulated after colistin treatment (Table 1 and Supplementary Table 1). It is possible that the ParR/ParS TCS positively regulates several genes involved in anaerobic respiration (*nirC*, *norC*, *norB*, *nosZ*, and *nosL*) (Fernández et al., 2010), however the reasons for activating the anaerobic respiratory chain in presence of colistin remain to be elucidated.

### Colistin Induced Up-Regulation of the MexT Regulon

Colistin caused a significant up-regulation of the PA14 genes *PA14_22420*, *PA14_22740*, *PA14_28410*, *mexF*, *mexE*, *PA14_32480, PA14_32490*, *PA14_39060*, *PA14_39420*, *PA14_41990, PA14_56620*, *PA14_56640*, and *PA14_64530* (Table 1 and Supplementary Table 1), all of which belong to the MexT regulon (Tian et al., 2009a; Hill et al., 2019). MexT is a transcriptional regulator of the LysR family known to control the expression of pathogenicity, virulence and antibiotic resistance determinants in *Pae* (Köhler et al., 1997a,b, 1999; Tian et al., 2009a; Huang et al., 2019). MexT regulates gene expression either directly through binding to the promoter region of distinct target genes, or indirectly through the activation of the MexEF-OprN efflux pump (Tian et al., 2009a,b; Olivares et al., 2012). Furthermore, MexT is a redox-responsive transcriptional activator implicated in diamide stress tolerance, in defense against the innate immune system-derived oxidant hypochlorous acid and against nitrosative stress (Fetar et al., 2011; Fargier et al., 2012; Farrant et al., 2020). Thus, the observed activation of MexT regulated genes might be a result of a defense mechanism being triggered against oxidative stress that arises as a consequence of colistin activity. As mentioned above, the denitrification pathway (*nir*, *nor*, and *nos* genes) was up-regulated in the presence of colistin (Table 1 and Supplementary Table 1), hence it is tempting to speculate whether this antibiotic can additionally inflict nitrosative stress to *Pae*. Moreover, Wang et al. (2013) showed that the deletion of *parR* and *parS* in *Pae* strain PAO1 negatively impacts the transcript abundance of genes belonging to the MexT regulon, without affecting the expression levels of *mexT*.

### Colistin Impacts the AlgU Regulon

Schulz et al. (2015) predicted that the primary regulon of the alternative sigma factor σ^22^ (AlgU or AlgT) in PA14 comprises 341 genes, while their mRNA profiling approach uncovered 222 genes that were down-regulated in an *algU* deletion- and up-regulated in an *algU* overexpressing strain, or *vice versa*. Our RNA-seq and Ribo-seq data sets show that colistin caused a change in expression at the transcriptional and/or the translational level of 141 out of those 222 AlgU-dependent genes (Supplementary Table 3). Envelope stress inducing agents cause proteolytic degradation of the AlgU anti-sigma factor MucA through regulated intramembrane proteolysis (RIP), which leads to the release of AlgU from the IM, and ultimately to the activation of the AlgU regulon (Wood et al., 2006; Damron and Goldberg, 2012). It is possible that the genes controlled by AlgU play a significant role in colistin susceptibility in *Pae*, as polymyxins have long been implicated in triggering envelope stress in Gram-negative Bacteria. As the transcription of *algU* itself was only slightly increased (2.65- fold) upon exposure to colistin (Supplementary Table 1), the regulation of the AlgU activity through RIP might explain the observed alterations in expression of the AlgU regulon. In view of our studies, we compared the susceptibility toward colistin of PA14 with an isogenic in frame *algU* deletion mutant. When compared with the PA14 WT strain, the minimal inhibitory concentration of colistin for PA14Δ*algU* was approximately fourfold reduced (Supplementary Figure 3), showing that the AlgU-dependent response counteracts the deleterious effects of colistin. In line with our observations, Murray et al. (2015) reported that a transposon insertion in *algU* affects the fitness of *Pae* in the presence of polymyxin B.

### Colistin Affects Multiple Efflux-Pump Genes

Besides the aforementioned MexXY-OprM, MexAB-OprM, MexEF-OprN, and MexGHI-OpmD efflux pumps, a strong colistin-dependent induction of the *mexC*D-*oprJ* and *mexJK* operons was observed (Table 1 and Supplementary Table 1). Expression of *mexCD-oprJ* was shown to be enhanced by polymyxin B in an AlgU-dependent manner (Fraud et al., 2008), whereas the MexJK efflux system has so far not been linked to polymyxin susceptibility.

### Tobramycin Down-Regulates Amino Acid Catabolism and Lower Tricarboxylic Acid Cycle Genes

The insertional inactivation of the genes encoding the Nuo and Nqr dehydrogenases was shown to increase tobramycin resistance of *Pae* (Schurek et al., 2008; Kindrachuk et al., 2011). It was hypothesized that their inactivation causes a reduction in the proton motive force and energy production, hence limiting the active uptake of tobramycin. The *nuo* and *nqr* genes were among the most down-regulated genes in the RNA-seq and Ribo-seq data after tobramycin treatment (Table 2 and Supplementary Table 1).

**TABLE 2:** Gene expression response of PA14 grown in the presence of tobramycin versus untreated control.

**Gene name**	**Gene ID**	**Gene product**	**RNA-seq**	**Ribo-seq**
			**FC^1^**	**FC**
**Energy metabolism and tricarboxylic acid cycle cycle**				
**PA14_06800**	PA14_06800	Hypothetical protein	**18.42**^2^	X^3^
**ldh**	PA14_19870	Leucine dehydrogenase	**–13.38**	–2.23
**PA14_19900**	PA14_19900	Pyruvate dehydrogenase E1 component subunit alpha	**–104.77**	–4.45
**pdhB**	PA14_19910	Pyruvate dehydrogenase E1 component. beta chain	**–94.26**	–2.53
**PA14_19920**	PA14_19920	Branched-chain alpha-keto acid dehydrogenase subunit E2	**–78.9**	X
**nqrA**	PA14_25280	Na(+)-translocating NADH-quinone reductase subunit A	6.06	ND^4^
**nqrB**	PA14_25305	Na(+)-translocating NADH-quinone reductase subunit B	ND	–4.6
**nqrC**	PA14_25320	Na(+)-translocating NADH-quinone reductase subunit C	–2.08	–2.66
**nqrD**	PA14_25330	Na(+)-translocating NADH-quinone reductase subunit D	–5.14	–3.25
**nqrE**	PA14_25340	Na(+)-translocating NADH-quinone reductase subunit E	–7.06	–2.51
**nqrF**	PA14_25350	Na(+)-translocating NADH-quinone reductase subunit F	–9.6	ND
**nuoN**	PA14_29850	NADH dehydrogenase subunit N	**–20.61**	**–13.71**
**nuoM**	PA14_29860	NADH dehydrogenase subunit M	**–24.8**	–3.99
**nuoL**	PA14_29880	NADH dehydrogenase subunit L	**–25.56**	–3.4
**nuoK**	PA14_29890	NADH dehydrogenase subunit K	–8	–6.97
**nuoJ**	PA14_29900	NADH dehydrogenase subunit J	–5.16	**–15.98**
**nuoI**	PA14_29920	NADH dehydrogenase subunit I	–9.55	**–13.6**
**nuoH**	PA14_29930	NADH dehydrogenase subunit H	**–10**	–4.88
**nuoG**	PA14_29940	NADH dehydrogenase subunit G	**–46.39**	–8.11
**nuoF**	PA14_29970	NADH dehydrogenase I subunit F	**–13.36**	–8.16
**nuoE**	PA14_29980	NADH dehydrogenase subunit E	**–13.55**	–5.35
**icd**	PA14_30190	Iisocitrate dehydrogenase	–3.09	ND
**sucD**	PA14_43940	Succinyl-CoA synthetase subunit alpha	–9.39	–3.27
**sucC**	PA14_43950	Succinyl-CoA synthetase subunit beta	–3.73	ND
**lpdG**	PA14_43970	Dihydrolipoamide dehydrogenase	**–16.47**	–5.26
**sucB**	PA14_44000	Dihydrolipoamide succinyltransferase	**–10.98**	–9.15
**sucA**	PA14_44010	2-oxoglutarate dehydrogenase E1	–4.27	–4.12
**PA14_53970**	PA14_53970	Aconitate hydratase	**–18.05**	–8.09
**Phenylalanine/Tyrosine catabolism**				
**fahA**	PA14_38530	Fumarylacetoacetase	**–30.98**	–2.32
**maiA**	PA14_38550	Maleylacetoacetate isomerase	**–48.44**	–3.57
**phhB**	PA14_53000	Pterin-4-alpha-carbinolamine dehydratase	**–14.76**	–2.47
**phhC**	PA14_53010	Aromatic amino acid aminotransferase	**–13.94**	–2.78
**Arginine catabolism**				
**arcD**	PA14_68300	Arginine/ornithine antiporter	**–49.29**	2.29
**arcA**	PA14_68330	Arginine deiminase	**–77.18**	–2.12
**arcB**	PA14_68340	Ornithine carbamoyltransferase	**–137.35**	–4.19
**Leucin/Valine/Isoleucin degradation and biosynthesis**				
**lpdV**	PA14_35490	Dihydrolipoamide dehydrogenase	**–104.5**	–6.56
**bkdB**	PA14_35500	Branched-chain alpha-keto acid dehydrogenase subunit E2	**–91.67**	–5.51
**bkdA2**	PA14_35520	2-oxoisovalerate dehydrogenase subunit beta	**–104.31**	–4.84
**bkdA1**	PA14_35530	2-oxoisovalerate dehydrogenase subunit alpha	–5.23	X
**gnyR**	PA14_38430	Regulatory gene of gnyRDBHAL cluster. GnyR	**–12.88**	ND
**gnyD**	PA14_38440	Citronelloyl-CoA dehydrogenase. GnyD	**–30.42**	–2.32
**gnyB**	PA14_38460	Acyl-CoA carboxyltransferase subunit beta	**–27.71**	X
**gnyH**	PA14_38470	Gamma-carboxygeranoyl-CoA hydratase	**–33.85**	–3.73
**gnyA**	PA14_38480	Alpha subunit of geranoyl-CoA carboxylase. GnyA	**–26.11**	X
**ilvA2**	PA14_47100	Threonine dehydratase	**24.7**	7.68
**Peptidoglycan biosynthesis**				
**ddl**	PA14_57320	D-alanine–D-alanine ligase	**–151.66**	–8.63
**murC**	PA14_57330	UDP-*N*-acetylmuramate–L-alanine ligase	**–78.75**	–9.99
**murG**	PA14_57340	UDPdiphospho-muramoylpentapeptide beta-*N*-acetylglucosaminyl transferase	**–44.2**	–8.27
**murD**	PA14_57370	UDP-*N*-acetylmuramoyl-L-alanyl-D-glutamate synthetase	**–46.8**	**–10.56**
**mraY**	PA14_57380	Phospho-*N*-acetylmuramoyl-pentapeptide-transferase	**–13.67**	–2.18
**murF**	PA14_57390	UDP-*N*-acetylmuramoylalanyl-D-glutamyl-2.6-diaminopimelate–D-alanyl-D-alanine ligase	**–11.12**	X
**murE**	PA14_57410	UDP-*N*-acetylmuramoylalanyl-D-glutamate–2. 6-diaminopimelate ligase	**–55.53**	–9.29
**rmlC**	PA14_68210	dTDP-4-dehydrorhamnose 3.5-epimerase	**–12.92**	–3.34
**Glycogen metabolism**				
**glgA**	PA14_36570	Glycogen synthase	–3.09	–5.06
**PA14_36580**	PA14_36580	Glycosyl hydrolase	**–19.03**	**–11.25**
**PA14_36590**	PA14_36590	4-alpha-glucanotransferase	**–34.5**	**–21.54**
**PA14_36605**	PA14_36605	Maltooligosyl trehalose synthase	**–27.97**	**–10.78**
**PA14_36620**	PA14_36620	Hypothetical protein	**–44.57**	**–10.58**
**PA14_36630**	PA14_36630	Glycosyl hydrolase	**–25.56**	–6.53
**glgB**	PA14_36710	Glycogen branching protein	**–38.34**	**–15.21**
**PA14_36730**	PA14_36730	Trehalose synthase	**–30.78**	**–16.68**
**PA14_36740**	PA14_36740	Hypothetical protein	–4.77	–5.04
**glgP**	PA14_36840	Glycogen phosphorylase	–5.98	–2.93
**PA14_36850**	PA14_36850	Hypothetical protein	–2.26	–2.9
**Pathogenicity and virulence**				
**tssL1**	PA14_00925	Hypothetical protein	**–17.71**	X
**tssk1**	PA14_00940	Hypothetical protein	–6.38	–4.32
**tssJ1**	PA14_00960	Lipoprotein	**–12.26**	–3.67
**PA14_00960**	PA14_00970	Hypothetical protein	**–33.07**	–2.84
**pilJ**	PA14_05360	Twitching motility protein PilJ	–2.63	ND
**pilK**	PA14_05380	Methyltransferase PilK	–2.59	ND
**chpA**	PA14_05390	ChpA	**–11.54**	–4.98
**PA14_05400**	PA14_05400	Methylesterase	**–20.38**	–3.72
**PA14_34000**	PA14_34000	HsiH3	**–23.95**	–5.54
**stk1**	PA14_42880	Stk1	–2.19	X
**stp1**	PA14_42890	Stp1	–5.56	–3.6
**PA14_42900**	PA14_42900	IcmF2	–3.59	–2.57
**PA14_42910**	PA14_42910	DotU2	**–11.99**	–3.08
**PA14_42920**	PA14_42920	HsiJ2	–7.75	–3.42
**PA14_42940**	PA14_42940	Lip2.2	**–20.14**	–3.56
**PA14_42950**	PA14_42950	Fha2	**–25.63**	–5.86
**PA14_42960**	PA14_42960	Lip2.2	**–77.39**	X
**PA14_42970**	PA14_42970	Sfa2	**–13.89**	–6.03
**PA14_42980**	PA14_42980	ClpV2	**–10.86**	–5.68
**PA14_42990**	PA14_42990	HsiH2	**–10.56**	–4.18
**PA14_43000**	PA14_43000	HsiG2	**–14.05**	–3.6
**PA14_43020**	PA14_43020	Hypothetical protein	**–10.04**	–3.57
**PA14_43030**	PA14_43030	HsiC2	–4.85	X
**flhB**	PA14_45720	Flagellar biosynthesis protein FlhB	–5.62	X
**fliR**	PA14_45740	Flagellar biosynthesis protein FliR	–8.13	X
**fliQ**	PA14_45760	Flagellar biosynthesis protein FliQ	**–10.6**	X
**fliP**	PA14_45770	Flagellar biosynthesis protein FliP	–6.41	X
**fliN**	PA14_45790	Flagellar motor switch protein	–2.05	X
**flgK**	PA14_50360	Flagellar hook-associated protein FlgK	**–75.19**	–3.47
**flgJ**	PA14_50380	Flagellar rod assembly protein/muramidase FlgJ	–9.17	–4.58
**flgI**	PA14_50410	Flagellar basal body P-ring protein	–6.22	X
**flgH**	PA14_50420	Flagellar basal body L-ring protein	–3.99	X
**flgG**	PA14_50430	Flagellar basal body rod protein FlgG	ND	2.13
**flgF**	PA14_50440	Flagellar basal body rod protein FlgF	ND	3.54
**pqsE**	PA14_51380	Quinolone signal response protein	**–80.7**	–2.98
**pqsD**	PA14_51390	3-oxoacyl-ACP synthase	**–90.01**	–5.86
**pqsC**	PA14_51410	PqsC	**–44.86**	–4.06
**pqsB**	PA14_51420	PqsB	**–20.71**	–2.34
**pqsA**	PA14_51430	PqsA	–3.73	X
**PA14_55780**	PA14_55780	Phosphate transporter	**–46.70**	X
**PA14_55790**	PA14_55790	Two-component sensor	**–15.49**	–2.70
**PA14_55800**	PA14_55800	Hypothetical protein	–2.21	X
**PA14_55810**	PA14_55810	Hypothetical protein	–2.73	–2.00
**PA14_55820**	PA14_55820	Two-component response regulator	**–25.16**	X
**PA14_55840**	PA14_55840	Hypothetical protein	**–84.76**	X
**PA14_55850**	PA14_55850	Hypothetical protein	**–68.52**	X
**PA14_55860**	PA14_55860	Pilus assembly protein	**–98.84**	X
**PA14_55880**	PA14_55880	Hypothetical protein	**–105.85**	X
**cpaF2**	PA14_55890	Hypothetical protein	**–111.85**	X
**PA14_55900**	PA14_55900	Type II secretion system protein	**–36.82**	–9.16
**PA14_55920**	PA14_55920	Hypothetical protein	**–15.84**	–2.00
**PA14_55930**	PA14_55930	Type II secretion system protein	–2.75	ND
**PA14_55940**	PA14_55940	Pilus assembly protein	–8.25	–7.96
**pilC**	PA14_58760	Type 4 fimbrial biogenesis protein pilC	–2.41	ND
**pilD**	PA14_58770	Type 4 prepilin peptidase PilD	**–11.48**	ND
**coaE**	PA14_58780	Dephospho-CoA kinase	ND	2.48
**fimU**	PA14_60280	Type 4 fimbrial biogenesis protein FimU	–2.46	ND
**pilW**	PA14_60290	Type 4 fimbrial biogenesis protein PilW	**–11.99**	X
**pilX**	PA14_60300	Type 4 fimbrial biogenesis protein PilX	–9.12	X
**pilY1**	PA14_60310	Type 4 fimbrial biogenesis protein PilY1	–4.06	ND
**pilE**	PA14_60320	Type 4 fimbrial biogenesis protein PilE	–3.43	–2.53
**PA14_65520**	PA14_65520	Hypothetical protein	**–21.34**	–3.44
**PA14_65540**	PA14_65540	Hypothetical protein	–4.26	X
**estA**	PA14_67510	Esterase EstA	**–11.29**	–2.17
**ABC transporters and Sha antiporter**				
**opuCA**	PA14_13580	ABC transporter ATP-binding protein	**–28.83**	–7.05
**opuCB**	PA14_13590	ABC transporter permease	–6	–4.83
**opuCD**	PA14_13600	ABC transporter substrate-binding protein	–4.73	–4.41
**nppA2**	PA14_41130	ABC transporter substrate-binding protein NppA2	–1.97	X
**nppB**	PA14_41140	Peptidyl nucleoside antibiotic ABC transporter permease NppB	**–10.23**	X
**nppC**	PA14_41150	Peptidyl nucleoside antibiotic ABC transporter permease NppC	**–30.15**	–3.71
**nppD**	PA14_41160	Peptidyl nucleoside antibiotic ABC transporter ATP-binding protein NppD	**–22.25**	–4.98
**fabI**	PA14_41170	NADH-dependent enoyl-ACP reductase	**–21.89**	–5.27
**phaG**	PA14_50680	ShaA	**–44.11**	–3.99
**phaF**	PA14_50690	ShaB	**–29.33**	–3.77
**phaE**	PA14_50700	ShaC	**–12.23**	–2.9
**phaD**	PA14_50710	ShaD	–7.08	–5.88
**phaC**	PA14_50720	ShaE	–8.48	–7.78
**dppC**	PA14_58450	Dipeptide ABC transporter permease DppC	**–13.03**	–3.55
**dppD**	PA14_58470	Dipeptide ABC transporter ATP-binding protein DppD	**–34.43**	**–10.45**
**dppF**	PA14_58490	Dipeptide ABC transporter ATP-binding protein DppF	**–21.5**	–4.19
**Transcription and translation**				
**tufB**	PA14_08680	Elongation factor Tu	**92.01**	3.09
**rplC**	PA14_08850	50S ribosomal protein L3	**27.97**	ND
**rplD**	PA14_08860	50S ribosomal protein L4	**23.35**	2.05
**tyrS**	PA14_10420	Tyrosyl-tRNA synthetase	**37.72**	**11.48**
**orf2**	PA14_12350	(dimethylallyl)adenosine tRNA methylthiotransferase	**23.15**	2.17
**rimM**	PA14_15980	16S rRNA-processing protein RimM	**55.59**	5.82
**trmD**	PA14_15990	tRNA (guanine-*N*(1)-)-methyltransferase	**52.24**	4.74
**rpsB**	PA14_17060	30S ribosomal protein S2	**106.77**	2.27
**deaD**	PA14_27370	ATP-dependent RNA helicase	**122.76**	2.02
**infC**	PA14_28660	Translation initiation factor IF-3	**12.93**	2.59
**yadB**	PA14_62510	Glutamyl-Q tRNA(Asp) synthetase	**34.45**	4.5
**yhbC**	PA14_62780	Hypothetical protein	**14.37**	ND
**smpB**	PA14_63060	SsrA-binding protein	**12.7**	2.36
**rpmE**	PA14_66710	50S ribosomal protein L31	**316.32**	ND
**prfH**	PA14_72200	Peptide chain release factor-like protein	**491.76**	5.65
**rnpA**	PA14_73420	Ribonuclease P	**164.46**	**16.33**
**Stringent response and toxin-antitoxin systems**				
**PA14_01510**	PA14_01510	Hypothetical protein	**25.78**	4.87
**PA14_01520**	PA14_01520	Hypothetical protein	**28.84**	4.71
**ndk**	PA14_14820	Nucleoside diphosphate kinase	6.47	ND
**obgE**	PA14_60445	GTPase ObgE	**15.46**	2.42
**vapI**	PA14_61840	Antitoxin HigA	9.67	5.8
**rnk**	PA14_69630	Nucleoside diphosphate kinase regulator	**11.23**	2.08
**spoT**	PA14_70470	Guanosine-3′.5′-bis(diphosphate) 3′-pyrophosphohydrolase	9.2	3.64

*^1^FC, fold-change, *p*-value ≤ 0.05. ^2^Genes with FC ≤ –10 or ≥10 are represented in bold. ^3^X, not efficiently translated in the control and antibiotic treated samples, Ribo-seq BaseMean ≤ 50. ^4^ND, not differentially expressed, –2 ≤ FC ≤ 2 and/or *p*-value ≥ 0.05.*

The catalytic activities of the isocitrate dehydrogenase Idh, the dihydrolipoamide succinyltransferase SucB and the aconitate hydratase PA14_53970 result in an increased NADH content and promote cellular respiration (Kohanski et al., 2007; Meylan et al., 2017). Upon tobramycin treatment, a down-regulation was observed for these genes of the lower part of the tricarboxylic acid (TCA) cycle (*idh*, *sucB*, and *PA14_53970*) (Table 2 and Supplementary Table 1). The diminished synthesis of enzymes of the lower part of TCA cycle upon tobramycin treatment is in agreement with a recent study, which suggested that *Pae* can bypass the decarboxylation steps of the cycle to reduce the NADH content, thus decreasing energy production. Growth of *Pae* on glyoxylate as a sole carbon source leads to the activation of this bypass, and consequently an increase in tobramycin resistance (Meylan et al., 2017).

Amino acid catabolism promotes the production of intermediate metabolites like fumarate, pyruvate, acetyl-CoA and α-ketoglutarate that fuel the TCA cycle, and thus could promote aminoglycosides uptake. Indeed, *Pae* responds to tobramycin by down-regulating genes encoding enzymes required for glycine and serine (*glyA2, gcvT2, sdaA*), phenylalanine (*pheB, pheC, maiA, fahA*), arginine (*arcABD*) and branched-chain amino acid (*bkdA1A2B-lpdV*, *gnyRBDHL* and *ldh*) catabolism (Table 2 and Supplementary Table 1).

Moreover, we also noted that the utilization pathways of D-alanine for peptidoglycan synthesis and transport (*ddl*, *mraY murC, murG, murD, murE*) and for glycogen metabolism (*PA14_36570*, *PA14_36580*, *PA14_36590*, *PA14_36605*, *PA14_36620*, *PA14_36630*, *glgB*, *PA14_36730*) were down-regulated after exposure to tobramycin (Table 2 and Supplementary Table 1).

### Tobramycin Affects the Expression of Functions Involved in Pathogenicity, Virulence and Transport

The comparative transcriptome and translatome analyses of PA14 treated with tobramycin uncovered a significantly reduced abundance of several virulence and pathogenicity related genes (Table 2 and Supplementary Table 1). The genes encoding the type II (*xcp* locus) and type VI (*tss, hsi*, and *hcp-1* locus) secretion systems, the quinolone-based quorum-sensing system (*pqs* genes) as well as the esterase (*estA*) were down-regulated in the RNA-seq and Ribo-seq data sets. Additionally, genes encoding for functions required for motility, attachment, pilus and fimbrial assembly (*PA14_55780-PA14_55940* including *tad* locus, *pil, flg, fli*, and *flh* genes) were primarily down-regulated at the transcriptional level. *Pae* may prevent tobramycin uptake through the down-regulation of genes of different secretion systems, as they are believed to be the entry gates for several structurally unrelated antimicrobial agents (Tzeng et al., 2005; Mulcahy et al., 2006). Although insertional inactivation of *pilZ* and *fimV* have been confirmed to confer low-level tobramycin resistance, it seems interesting to assess the contribution of other motility and attachment genes to aminoglycoside permeability, such as those of the *tad* locus (Schurek et al., 2008).

The genes encoding ABC transporters such as the dipeptide permease Dpp, involved in the uptake of kasugamycin in *E. coli* (Shiver et al., 2016), the permease Npp, which plays a role in the translocation of the uridyl peptide antibiotic pacidamycin in *Pae* (Luckett et al., 2012; Pletzer et al., 2015) and the ATP-binding protein OpuC, showed a tobramycin-dependent down-regulation in both, the RNA-seq and Ribo-seq data. Moreover, the Na^+^/H^+^ antiporter *pha* (*sha*) operon important for the homeostasis of monovalent cations (Kosono et al., 2005) was also strongly down-regulated after exposure to tobramycin (Table 2 and Supplementary Table 1). The Na^+^/H^+^ Sha antiporter shows similarity to the membrane subunits of the respiratory Nuo complex and could therefore be of interest for further analysis with regard to its potential impact on the proton motive force and thus aminoglycoside uptake (Mathiesen and Hägerhäll, 2003).

### Tobramycin Impacts the Abundance of Genes Involved in Translation

Tobramycin promotes mistranslation, stop codon read-through and ribosome stalling (Aboa et al., 2002; Thompson et al., 2002; Harms et al., 2003; Vioque and Cruz, 2003). A number of genes related to translation were strongly up-regulated in the RNA-seq and Ribo-seq data, including the genes encoding translation initiation factor 3 (*infC*), ribosomal proteins (*rpsB, rplC, rplD*), a putative ribosomal maturation factor (*yhbC*), elongation factor EF-Tu (*tufB*) and ribonuclease P (*rnpA*) (Table 2 and Supplementary Table 1).

tRNA modifications can play an important role in the modulation of antibiotic resistance by regulating translational processes (Chopra and Reader, 2015). The genes encoding the tRNA methyltransferases TrmD, Orf2, and RimM were significantly up-regulated by tobramycin (Table 2 and Supplementary Table 1). TrmD is involved in the m1G37 methylation of proline tRNA (Gamper H.B. et al., 2015; Gamper H. et al., 2015), and recent studies in *E. coli* and *S. enterica* revealed that the translation of several membrane-associated proteins is controlled by m1G37 methylation at proline codons near the start of their respective open reading frames. TmrD deficient strains exhibit a decrease in membrane protein content resulting in a higher susceptibility to aminoglycosides (Masuda et al., 2019).

*Trans*-translation is a process adopted by the cell to rescue stalled ribosomes that requires the specialized tmRNA SsrA and the small accessory protein SmpB (Withey and Friedman, 2003; Haebel et al., 2004). Pathogenic bacteria lacking this system display an enhanced sensitivity toward aminoglycosides (de la Cruz and Vioque, 2001; Aboa et al., 2002; Morita et al., 2006). In this study, several genes encoding effectors of stalled ribosome rescue (*PA14_72200*, tmRNA *ssrA* and *smpB* encoding an accessory protein) were up-regulated after tobramycin exposure (Table 2 and Supplementary Table 1).

Furthermore, the gene encoding ObgE, a conserved ribosomal associated GTPase with unknown function (Verstraeten et al., 2015), was up-regulated upon tobramycin treatment (Table 2 and Supplementary Table 1). Verstraeten et al. (2015) showed that over-expression of *obgE* confers tobramycin and ofloxacin tolerance to *Pae* and *E. coli*. They further reported that in *E. coli* Obg-mediated tolerance requires activation of the type I *hokB-sokB* TA system. Although a *hokB* ortholog is not present in *Pae*, we found multiple genes associated with type II TA systems including HigA (VapI) and ParE-ParD (*PA14_01510-PA14_01520*) that are up-regulated in the presence of tobramycin (Table 2 and Supplementary Table 1).

### Comparison With Previous Transcriptome Studies

When compared with previous *Pae* transcriptome studies performed in the presence of polymyxins and tobramycin (Cummins et al., 2009; Fernández et al., 2010; Kindrachuk et al., 2011; Murray et al., 2015; Han et al., 2019; Ben Jeddou et al., 2020) this study revealed a larger number of de-regulated genes. Despite some variances, overlaps concerning de-regulated genes exist. The *arn* operon, the *speD2-speE2* (*PA14_63110- PA14_63120*) genes, the *mexAB-oprN*, the *mexC*, the *mexXY* (*PA14_38395* and *amrB*), the *galU*, the *cprA* (*PA14_43311*) and the genes of unknown function *PA2358* (*PA14_34170*), *PA1797* (*PA14_41280*), *PA14_41290* and *PA4782* (*PA14_63220*) were also previously found to be de-regulated in response to polymyxins or in *Pae* strains harboring mutations that impact polymyxin resistance (Cummins et al., 2009; Fernández et al., 2010; Murray et al., 2015; Han et al., 2019; Ben Jeddou et al., 2020) (Table 1 and Supplementary Table 1). Kindrachuk et al. (2011) reported that bacteriostatic and bactericidal concentrations of tobramycin stimulate the expression of several heat shock genes and genes encoding transcriptional regulators, whereas genes involved in energy metabolism (i.e., *nuo, nqr*, and *suc* genes), motility and attachment (i.e., *pil* and *flg* genes) were down-regulated. Our RNA-seq and Ribo-seq results closely mirror these previous findings (Figure 4 and Supplementary Table 1).

The transcriptional repression of iron homeostasis- (i.e., *has*, *pvd*, *pch*, *fpt*, *fpv*) and sulfonate utilization genes (*ssu*) (Tralau et al., 2007) and an up-regulation of the denitrification pathway genes (*nir*, *nor*, *nos*) upon exposure to polymyxin B has been reported for PA14 grown in Mueller–Hinton broth (Ben Jeddou et al., 2020). However, this study did not reveal a positive effect on expression of oxidative stress response genes by polymyxin B. On the other hand, transcription of *PA14_24360, ahpF*, and *ahpB* was seemingly induced when PA14 was exposed to synthetic antimicrobial peptide dendrimers (Ben Jeddou et al., 2020).

## Conclusion

In this study, SCFM was used for culturing *Pae*, a medium that approximates the environment in the lungs of CF patients (Palmer et al., 2007). To the best of our knowledge, no other gene profiling study has offered a more comprehensive view of *Pae*’s cellular responses to colistin and tobramycin, and especially under these culturing conditions.

**FIGURE 5:**
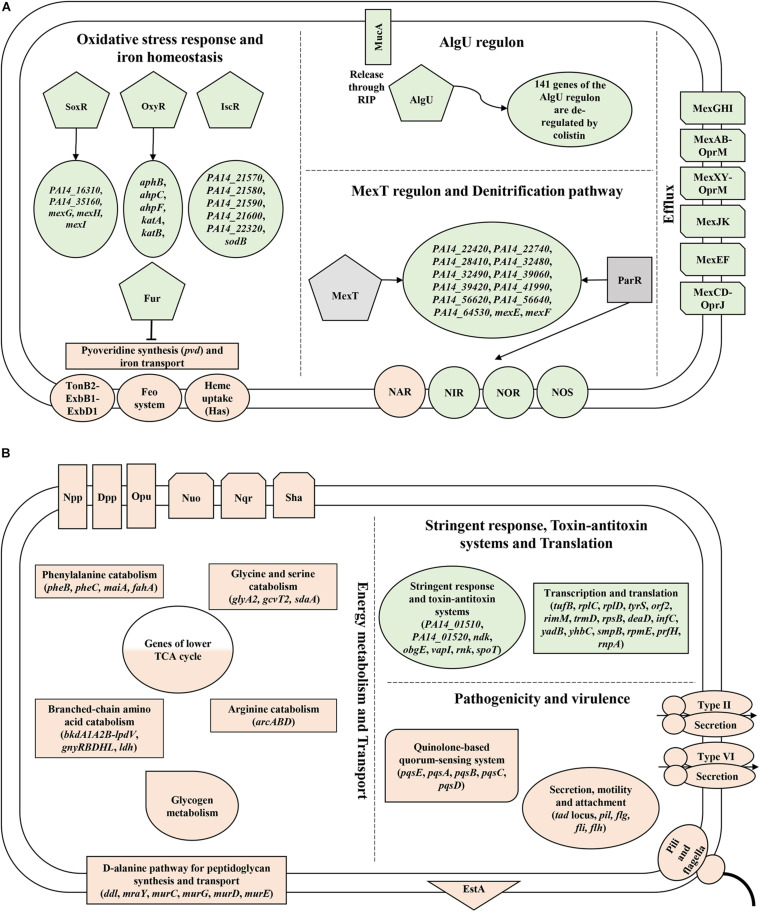
Depiction of novel functions/pathways revealed in this study that are de-regulated upon **(A)** colistin and **(B)** tobramycin treatment. Major genes/pathways that are down-regulated and up-regulated based on the RNA-seq and/or Ribo-seq data are highlighted in rose and green, respectively. Positive- and negative regulation of gene expression is denoted by arrows and blocked lines, respectively. RIP - regulated intramembrane proteolysis.

Although the potential of colistin to instigate ROS production in *Pae* is known, this study revealed for the first time its impact on the expression of distinct oxidative stress response genes. Moreover, the study disclosed a colistin-dependent de-regulation of the AlgU regulon and an up-regulation of the MexT regulon taking on a previously undescribed roles in defense against polymyxin antibiotics (Figure 5).

The transcriptome and translatome studies further indicated that the expression of multiple amino acid catabolism genes, lower TCA cycle genes, type II and VI secretion system genes and genes involved in motility and attachment are rewired in response to tobramycin, presumably to reduce drug uptake. Moreover, we discussed that the adverse effects of tobramycin on translation are countered through the expression of functions involved in stalled ribosome rescue, tRNA methylation and type II TA systems. These findings might aid toward the optimization of strategies to increase the efficacy of these last resort drugs against *Pae* (Figure 5).

Moreover; our results implicate a number of hypothetical genes of unknown function in colistin and tobramycin resistance (Supplementary Table 1). Deciphering their roles could be the basis for future research to elucidate additional mechanisms of action and resistance to colistin and tobramycin.

## Data Availability Statement

The datasets presented in this study can be found in online repositories. The names of the repository/repositories and accession number(s) can be found in the article/ Supplementary Material.

## Author Contributions

ACS, BL, ES, and UB conceived and designed the experiments. ACS and BL performed the experiments. ACS, BL, FA, MW, and UB analyzed the data. ACS, BL, and UB wrote the manuscript. All the authors contributed to the article and approved the submitted version.

## Conflict of Interest

The authors declare that the research was conducted in the absence of any commercial or financial relationships that could be construed as a potential conflict of interest.

## Footnotes

1 http://www.csf.ac.at

2 http://www.bioinformatics.babraham.ac.uk/projects/fastqc/

## Supplementary Material

Supplementary Figure 1 Activity of **(A)** 8 μg/ml colistin and **(B)** 64 μg/ml tobramycin on growth of *Pseudomonas aeruginosa* PA14. The arrows represent the points at which antibiotics were added to growing cultures (OD_600_ of 1.7). Error bars indicate standard deviations obtained from two biological replicates.

Supplementary Figure 2 Superimposition of the **(A)** *tonB2-exbB1-exbD1*, **(B)** *feo*, **(C)** *has*, and **(D)** *pvd* genes with the ribosome profiling data. In pink – open reading frames (ORF) of corresponding genes located on the negative strand of PA14 genomic DNA; in orange – ORFs of corresponding genes located on the positive strand of PA14 genomic DNA; in light green – mapped ribosomal footprints obtained from control samples, in dark green – mapped ribosomal footprints obtained from colistin treated samples.

Supplementary Figure 3 Increased susceptibility of PA14Δ*algU* toward colistin. **(A)** The microdilution assay was performed in duplicate with strains PA14 and PA14Δ*algU*, aerobically grown in SCFM medium to an OD_600_ of ∼2.0. Then, 0.5 ml of the culture was mixed with 1.5 ml of SCFM medium, containing serial dilutions of colistin (concentration 4–64 μg/ml). The cultures were shaken at 37°C for 20 h and the pictures were taken. The minimal inhibitory concentrations (MICs; marked by red edging) correspond to the lowest concentration of colistin that visibly impeded growth. Control, no colistin added. **(B)** Graphical representation of the results shown in **(A)**. The outcome of the duplicate assay was identical.

Supplementary Table 1 RNA-seq and Ribo-seq differential gene expression analysis of *Pae* treated with colistin or tobramycin versus untreated control.

Supplementary Table 2 Changes in transcript and ribosomal footprint abundance of genes which contribute to polymyxin and aminoglycoside resistance/susceptibility in *Pae* after exposure to colistin and tobramycin.

Supplementary Table 3 Changes in transcript and ribosomal footprint abundance of genes belonging to the AlgU regulon in *Pae* (Schulz et al., 2015) in the presence of 8 μg/ml colistin.
