# Supplementary material for: Gene Expression Profiling of Pseudomonas aeruginosa Upon Exposure to Colistin and Tobramycin
Source: Front Microbiol. 2021 Apr 30;12:626715. doi: 10.3389/fmicb.2021.626715 (PMC8120321; doi:10.3389/fmicb.2021.626715)
Supplement: Supplementary Figure 1 — Activity of (A) 8 μg/ml colistin and (B) 64 μg/ml tobramycin on growth of Pseudomonas aeruginosa PA14. The arrows represent the points at which antibiotics were added to growing cultures (OD600 of 1.7). Error bars indicate standard deviations obtained from two biological replicates. [file Data_Sheet_1.pdf]

Supplementary Material

Gene expression profiling of *Pseudomonas aeruginosa* upon exposure to colistin and tobramycin

Anastasia Cianciulli Sesso[†], Branislav Lilić[†], Fabian Amman, Michael T. Wolfinger, Elisabeth Sonnleitner and Udo Bläsi[*]

***Correspondence:**

Udo Bläsi
udo.blaesi@univie.ac.at

SUPPLEMENTARY TEXT

Construction of strain PA14Δ*algU*

To construct the in frame *algU* deletion strain PA14Δ*algU*, the allelic exchange technique was used as previously described (Hmelo et al., 2015). Briefly, two PCR products were obtained using primer pairs E179 (5'-AAA AA**G AAT TC**G CTG CCT TCC TCG TGG TT-3') / F179 (5'-TGG CAT TTG CCG CTG TGT CAG AAA GCT CCT CTT CGA ACC T-3') and G179 (5'-TGA CAC AGC GGC AAA TGC-3') / H179 (5'-AAA AA**G GAT CC**C TCG TAG ACG AAG GTG CCT-3') and chromosomal DNA of PA14, respectively. The resulting 761-bp upstream and 779-bp downstream fragments were then annealed and used as template for a second overlapping PCR with primers E179 and H179. The resulting PCR amplicons were cleaved with BamHI and EcoRI and ligated into the corresponding sites of plasmid pEXG2 (Rietsch et al., 2005). The generated plasmid pEXG2Δ*algU* was subsequently mobilized into PA14 WT strain with the aid of *Escherichia coli* strain S17-1 (Simon et al., 1983), and finally chromosomally integrated through selection for gentamycin resistance. Excision of the vector by a second crossover event was achieved by selection for sucrose insensitive cells, as the pEXG2 vector encodes the *Bacillus subtilis sacB* gene, the product of which – levan sucrose – renders *Pae* sensitive to sucrose.

Supplementary References

Hmelo, L. R., Borlee, B. R., Almblad, H., Love, M. E., Randall, T. E., Tseng, B. S., et al. (2015). Precision-engineering the *Pseudomonas aeruginosa* genome with two-step allelic exchange. *Nat. Protoc.* 10, 1820–1841. doi:10.1038/nprot.2015.115.

Rietsch, A., Vallet-Gely, I., Dove, S. L., and Mekalanos, J. J. (2005). ExsE, a secreted regulator of type III secretion genes in *Pseudomonas aeruginosa*. *Proc. Natl. Acad. Sci. U. S. A.* 102, 8006–8011. doi:10.1073/pnas.0503005102.

Simon, R., Priefer, U., and Pühler, A. (1983). A Broad Host Range Mobilization System for *in vivo* Genetic Engineering: Transposon Mutagenesis in Gram Negative Bacteria. *Bio/Technology* 1, 784–791. doi:10.1038/nbt1183-784.

SUPPLEMENTARY FIGURES

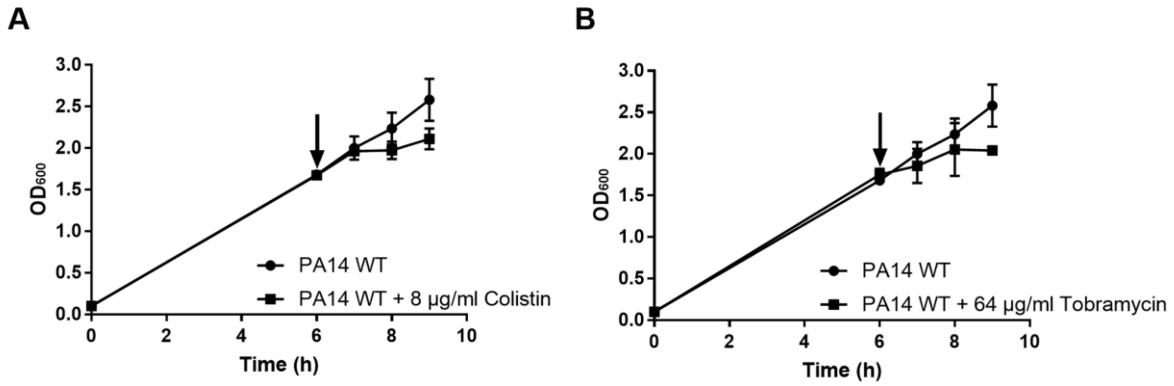

Supplementary Figure 1. Activity of **(A)** 8 µg/ml colistin and **(B)** 64 µg/ml tobramycin on growth of *Pseudomonas aeruginosa* PA14. The arrows represent the points at which antibiotics were added to growing cultures (OD_{600} of 1.7). Error bars indicate standard deviations obtained from two biological replicates.

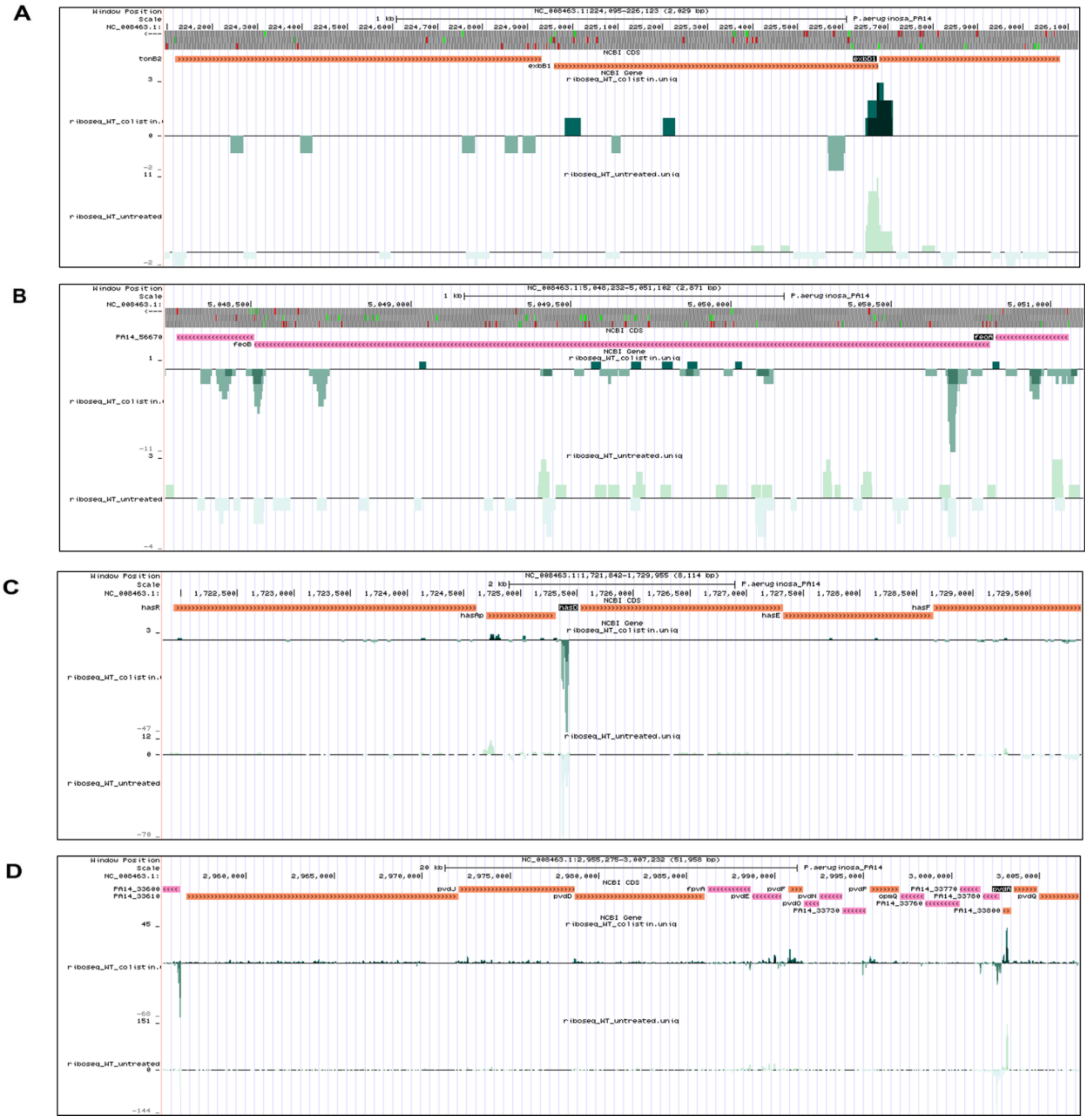

Supplementary Figure 2. Superimposition of the **(A)** *tonB2-exbB1-exbD1*, **(B)** *feo*, **(C)** *has* and **(D)** *pvd* genes with the ribosome profiling data. Legend: in pink – open reading frames (ORF) of corresponding genes located on the negative strand of PA14 genomic DNA; in orange - ORFs of corresponding genes located on the positive strand of PA14 genomic DNA; in light green - mapped ribosomal footprints obtained from control samples, in dark green - mapped ribosomal footprints obtained from colistin treated samples.

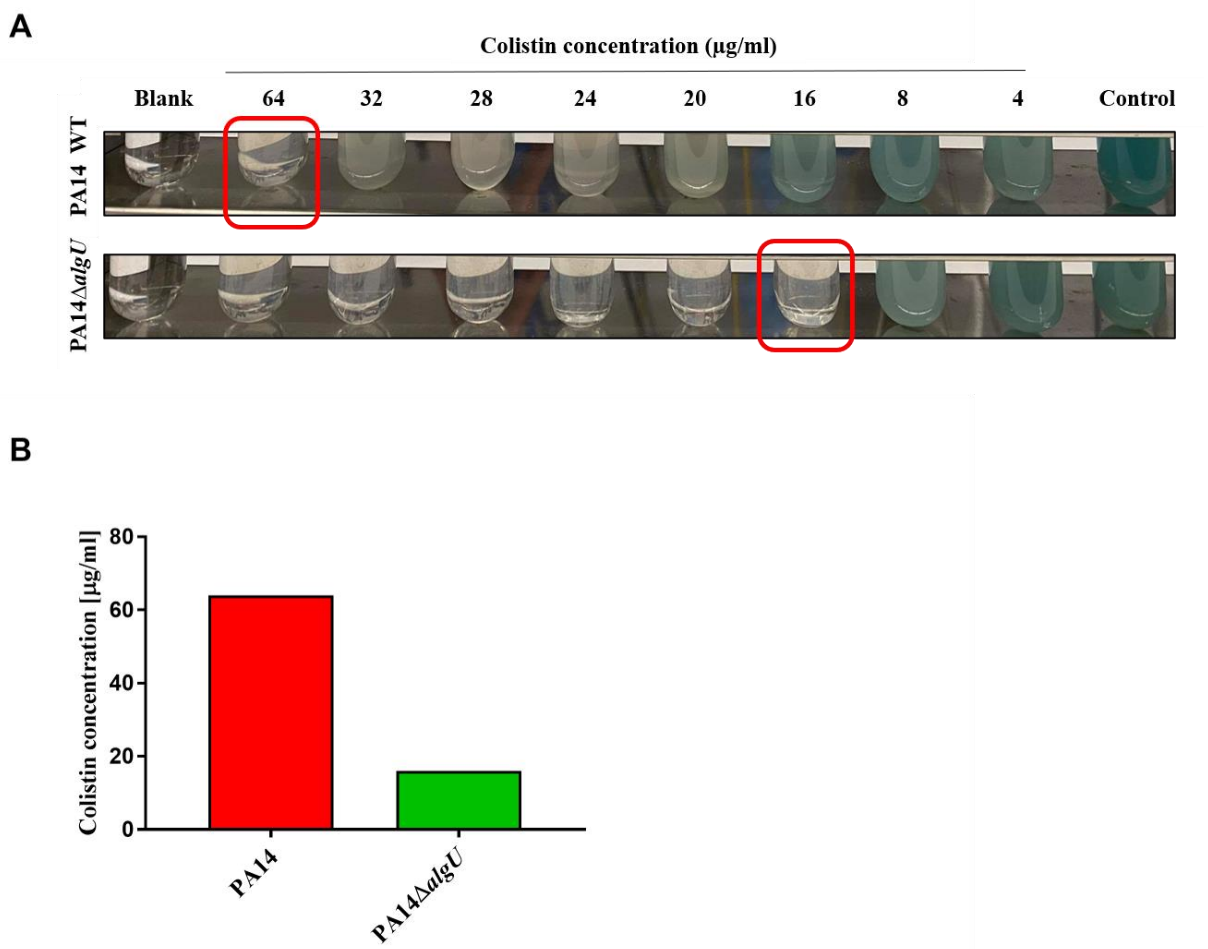

Supplementary Figure 3. Increased susceptibility of PA14Δ*algU* towards colistin. **(A)** The microdilution assay was performed in duplicate with strains PA14 and PA14Δ*algU*, aerobically grown in SCFM medium to an OD_{600} of ~2.0. Then, 0.5 ml of the culture was mixed with 1.5 ml of SCFM medium, containing serial dilutions of colistin (concentration 4 to 64 μg/ml). The cultures were shaken at 37°C for 20 h and the pictures were taken. The minimal inhibitory concentrations (MICs; marked by red edging) correspond to the lowest concentration of colistin that visibly impeded growth. Control, no colistin added. **(B)** Graphical representation of the results shown in **(A)**. The outcome of the duplicate assay was identical.
